# Draft Genome Sequence of *Pseudomonas* sp. Strain Ep R1 Isolated from *Echinacea purpurea* Roots and Effective in the Growth Inhibition of Human Opportunistic Pathogens Belonging to the *Burkholderia cepacia* Complex

**DOI:** 10.1128/genomeA.00351-17

**Published:** 2017-05-18

**Authors:** Valentina Maggini, Luana Presta, Elisangela Miceli, Marco Fondi, Emanuele Bosi, Carolina Chiellini, Camilla Fagorzi, Patrizia Bogani, Vincenzo Di Pilato, Gian Maria Rossolini, Alessio Mengoni, Fabio Firenzuoli, Elena Perrin, Renato Fani

**Affiliations:** a Department of Biology, University of Florence, Florence, Italy; b Department of Experimental and Clinical Medicine, University of Florence, Florence, Italy; c Center for Integrative Medicine, Careggi University Hospital, University of Florence, Florence, Italy; d Department of Surgery and Translational Medicine, University of Florence, Florence, Italy; e Clinical Microbiology and Virology Unit, Careggi University Hospital, Florence, Italy

## Abstract

In this announcement, we detail the draft genome sequence of the *Pseudomonas* sp. strain Ep R1, isolated from the roots of the medicinal plant *Echinacea purpurea*. The elucidation of this genome sequence may allow the identification of genes associated with the production of antimicrobial compounds.

## GENOME ANNOUNCEMENT

Endophytic bacterial communities inhabiting the rhizosphere or internal tissues of the medicinal plants (MPs) may contribute to the therapeutic properties of these plants (1). Here we report on the draft genome sequence of* Pseudomonas* sp. strain Ep R1, a strain isolated from the roots of *Echinacea purpurea*, an MP with immunomodulant, antiviral, and antimicrobial activity (2). The *E. purpurea* bacterial endophytes were isolated and molecular and phenotypic characterizations were conducted (3). In particular, *Pseudomonas* sp. Ep R1 showed the ability to inhibit the growth of other *E. purpurea* endophytes (4) and of cystic fibrosis bacterial pathogens belonging to the *Burkholderia cepacia* complex (5). Moreover, it has been demonstrated to be highly (50 μg/ml) resistant to chloramphenicol and streptomycin (6).

The genome sequence of *Pseudomonas* sp. Ep R1 was determined by a 2- × 300-bp paired-end approach using the MiSeq sequencing system (Illumina Inc., San Diego, CA). A total of 1,148,852 paired-end reads were obtained, representing approximately 100× coverage of the whole genome. *De novo* assembly was performed using SPAdes 2.3 (7), which generated 363 contigs. Contigs with length less than 2,000 bp were discarded. The remaining contigs were used for a multidraft-based analysis using genome sequences of 13* Pseudomonas* strains retrieved from the NCBI database (*P. aeruginosa* PAO1, *P. alkylphenolia* KL28, *P. denitrificans* ATCC 13867, *P. entomophila* L48, *P. fluorescens* F113, *P. fulva* 12-X, *P. knackmussii* B13, *P. mendocina* ymp, *P. protegens* CHA0, *P. putida* KT2440, *P. resinovorans* NBRC, *P. stutzeri* CGMCC, and *P. syringae* pv. tomato DC3000) through MeDuSa scaffolder (8). The final version of the draft genome assembly of *Pseudomonas* sp. Ep R1 is 6,797,087 bp long and embeds 158 contigs (the longest of which is 1,954,067 bp long). The G+C content is 65.5%, similar to that of other *Pseudomonas* genomes sequenced so far. Automated annotation of the *Pseudomonas* sp. Ep R1 draft genome sequence using the NCBI Prokaryotic Genome Annotation Pipeline detected 6,001 protein-coding genes, 67 RNA-coding genes (7 complete rRNAs, 56 tRNAs, 4 noncoding RNAs [ncRNAs]), and 173 pseudogenes.

Genes involved in the biosynthesis of secondary metabolites with antimicrobial activity were searched. The analysis was performed within an antiSMASH shell (9), which revealed that the *Pseudomonas* Ep R1 genome harbors 6 clusters involved in the biosynthesis of streptomycin, stenothricin, pimaricin, type 3 polyketide synthase (T3PKS), siderophore (desferrioxamine B), and nonribosomal peptide synthetase (NRPS) (amychelin). Moreover, the genome sequence was analyzed through CARD (10), which led to the identification of several genes (*mexABEJKMNW*, *omrMN*, *katG*, *triC*, *mfd*, and *mdtC*) putatively involved in antibiotic resistance, some conferring specific resistance to fluoroquinolone, mupirocin, beta-lactam, aminocoumarin molecules, and others involved in regulatory or inactivating systems and efflux pumps.

### Accession number(s).

This whole-genome shotgun project has been deposited in GenBank under the accession no. MWTQ00000000. The version described in this paper is the version MWTQ00000000.1.
